# Dissection and hemostasis with hydroxilated polyvinyl acetal tampons in open thyroid surgery

**DOI:** 10.1186/1750-1164-1-3

**Published:** 2007-02-20

**Authors:** Gianlorenzo Dionigi, Luigi Boni, Francesca Rovera, Renzo Dionigi

**Affiliations:** 1 Department of Surgical Sciences, University of Insubria, Viale Borri 57, 21100 Varese, Italy

## Abstract

**Background:**

The essential objectives for thyroidectomy are: avoidance of injury to the recurrent laryngeal nerves, conservation of the parathyroid glands, an accurate haemostasis and an excellent cosmesis. In the last 10 years major improvements and new technologies have been proposed and applied in thyroid surgery; among these mini-invasive thyroidectomy, regional anaesthesia and intraoperative neuromonitoring, and new devices for achieving dissection and haemostasis. Minor bleeding from small vessels could be a major complication in thyroid surgery. The purpose of ligating vessels is to maintain the surgical site free from an excess of blood and reduce blood loss in the patient.

**Materials and methods:**

Hydroxylated polyvinyl acetal tampons (HPA) are made by a synthetic, open cell foam structure able to absorb fluids up to 25 times the initial weight. We tested their efficacy for small bleeding control and tissue dissection during several thyroid procedures.

**Results:**

HPA tampons have been found extremely useful to absorb blood coming from minor and diffuse loss, helping to control bleeding by a combined action of fluid absorption and local compression. The porous design of the tampon allows the use of the suction device right through the tampon itself. Thanks to the initial mildly hard consistency, we also used HPA tampons as dissecting instruments.

**Conclusion:**

In our experience the use of HPA tampons resulted extremely efficient for minor bleeding control, fluids removal and tissue dissection during thyroid surgery.

## Background

New technologies have been proposed and applied in thyroid surgery, such as the mini-invasive video-assisted thyroidectomy (MIVAT) and the intraoperative monitoring of recurrent laryngeal nerve (RLN) [1,2]. In general the essential objectives for thyroidectomy are: conservation of the parathyroid glands, avoidance of injury to RNL, an accurate hemostasis and an excellent cosmesis. The thyroid has a rich blood supply. Each must be securely occluded and divided to perform a safe and expeditious operation [3]. Theodor Kocher is credited with refining the technique of thyroidectomy and reducing the incidence of postoperative hemorrhage [3]. It is difficult to estimate the real impact of bleeding, as main cause of intra-operative accidental lesions of vital structures as RLN [3]. However any surgeon who has routinely been practising thyroid surgery, knows that even minor bleeding may greatly compromise the view of surgical field and lead to severe difficulties in identifying the anatomical structures. Furthermore, management of abnormal bleeding exposes the patient to the morbidity of re-operation. In mini-invasive thyroidectomy intraoperative bleeding is a frequent cause for conversion to open technique [2]. Several devices and techniques, coming from general surgery, are commonly used to control bleeding, during thyroid surgery. Haemostasis in thyroid surgery is achieved by means of conventional clamp-and-tie technique, diathermy, and haemostatic clips and, more recently, by ultrasonic coagulating-dissection and electrothermal bipolar vessel sealing systems. We tested hydroxylated polyvinyl acetal tampons (HPA), their efficacy for small bleeding control and tissue dissection during several thyroid procedures.

## Materials and methods

### Tampons

We tested the use of hydroxylated polyvinyl acetal (HPA) tampons (Merocel™ – Medtronic Xomed, Jacksonville, Florida, USA) for minor bleeding control, fluid absorption and dissection during thyroid procedures. Hydroxylated polyvinyl acetal tampons are made of synthetic molecule obtained by "foaming at open cell" a fully biocompatible nonirritating polymer. This manufacturing procedure allows the generation of a cellular net made by pores, which are joined together in order to prevent breaks or lost of fibers. They are designed in different shapes, forms and sizes. Specific test demonstrated that HPA tampons are fully biocompatible and able to reduce bacterial grown. Their surface is smooth and they do not stick to tissues. They have an initial, mildly hard, firmness that allows their use as blunt dissection devices. Once in contact with fluids, the polymeric structure provides great absorbing capacity (up to 25 times the original weigh) within a small volume of material. Merocel™ tampons are routinely used for removal of unwanted fluids from the operative field in general, neurosurgical operations and for nasal packing in the treatment of epistaxis [4,5,8-10].

### Clinical experience

From August 2004 till December 2005, HPA tampons have been used, on demand, in 50 different thyroid procedures (Table 1, 2).

**Table 1:** Clinical experience with HPA tampons

**Thyroid procedure with HPA tampon**	**Number**
Total thyroidectomy	43
Emithyroidectomy	7
TOTAL	50

**Table 2:** Final pathology

**Thyroid procedure with HPA tampon**	**Number**
Nontoxic Goiter	31
Graves' Disease	15
Differentiated Thyroid Carcinoma	4
TOTAL	50

In 47/50 (94%) cases only one tampon was required, in 3/50 (6%) patients required 2 tampons. This fact is mainly related to the thyroid volume and length of the procedure. The present study did not include cervical lymph node dissections or miniinvasive thyroid procedures.

## Results

HPA has been used for both benign and malignant thyroid disease, for hemithyroidectomy as well as for total thyroidectomy (Table 1, 2). HPA tampons have been found extremely useful to absorb blood coming from minor and diffuse losing, caused by dissection of tissues, adhesions and small vessel. Once in contact with the bleeding area, HPA tampons are able to expand quickly, helping to control bleeding by a combined action of fluid absorption and local compression, that facilitates platelets aggregation. Once expanded, the tampon becomes soft and a traumatic and it can be used as gentle retracting and dissecting instrument reducing the risk of accidental damaging.

**Figure 1:**
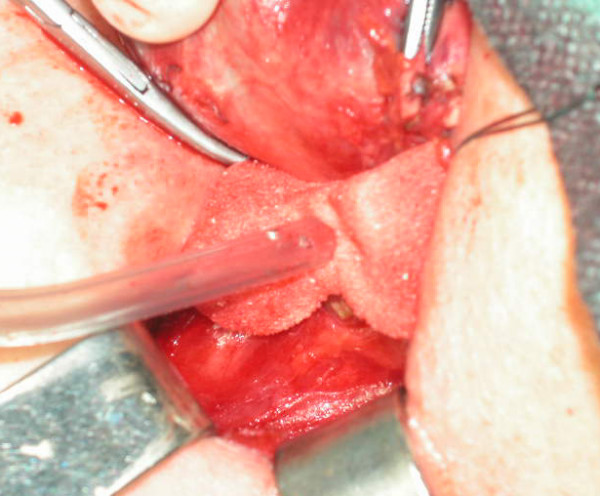
Minor bleeding control using HPA tampon, during thyroidectomy. The porous design of the tampon allows performing an effective aspiration of blood and unwanted fluid, by positioning the tampon directly on the bleeding surface (for example, close to RNL) and using the suction device right through the tampon itself.

In a previous laboratory test we found than 1 cm^2 ^of HPA tampon was able to absorb a significantly greater amount of blood than the same surface of standard surgical swab; it has also been found that HPA tampons were able to absorb fluids up to 25 time their initial weight [4]. The porous design of the tampon allows performing an effective aspiration of blood and unwanted fluid, by positioning the tampon directly on the bleeding surface (for example close to RNL) and using the suction device right through the tampon itself (Fig. 1). Thanks to the initial hard consistency, we also used HPA tampons as dissecting instruments during thyroidectomy. This resulted extremely useful for the dissection of the thyroid gland from the trachea and muscles, to identify and isolate the RLN or for blunt dissection and taking down of adhesions. In total thyroidectomy, the tampon was left in situ while performing the controlateral emithyroidectomy. Furthermore, opposite to standard pledgets, HPA tampons are totally fibers free and extremely resistant to strong tractions. Their cost is also fairly low (less than 10€ per tampon).

No postoperative mortality or morbidity in these short series was observed. None of the patients showed evidences of allergic reactions or intolerance; no accidental rupture or intra-abdominal lost of tampons were reported.

## Conclusion

The use of local haemostatic devices is spread in general surgery to assist in the control of capillary, venous, small arterial haemorrhage and "oozing" bleeding when legation or other conventional methods of control are impractical or ineffective. New modalities for the haemostasis and dissection in thyroid surgery include clips, tampons, harmonic scalpel, fibrin sealant, electrothermal bipolar vessel sealing system and gauzes. The effect of a combination of new haemostatic devices on the treatment of dissection and bleeding in thyroid surgery aim to improve primary and secondary haemostasis rates and the safety of treatment [6]. There are no data in Literature of the use of HPA in thyroid surgery in particular open thyroidectomies. Their easy application, low cost, and significant blood-loss reduction make these agents attractive also for thyroid surgery. The use of the haemostats has been approved by the U.S. Food and Drug Administration. In our experience HPA have been documented to be a safe device in open surgery.

During thyroid procedures the tampons are cut to the appropriate size for the placement, with a sterile technique in the begining phases of surgery. The device may be adjusted it in the irregular surfaces of the dissecting area between the trachea and the strap muscles thus protecting the RLN from the suctioning drainage. The haemostat, by swelling, may exert pressure resulting in paralysis and/or nerve damage: the hypothesis that these surgical medicaments placed in close proximity to the RLN could be responsible for some postoperative disturbances and that any alteration in nerve function that occurs postoperatively is usually attributed to the trauma of the surgical procedure rather than the medicament is quite difficult to determine. The surgical sponge has a pH of 6.5 ± 1.0 and should not be placed adjacent to neural tissue. Despite this advice it is often placed in direct contact with neural tissue, particularly in neurosurgery. Loescher and Robinson examined the response of tissue to different haemostatics agents left in situ in particular the neural function was reported [7]. The immediate effect of the haemostat on neural function was rare and variable, responsible for some postoperative disturbances, but the effects of these haemostats on neural function were only temporary. We suggest in patients with haemorrhagic disorders, those requiring oral anticoagulant therapy, with persistent major bleeding after primary haemostatic measures during thyroid surgery, hemostasis can be achieved through these new devices. These new devices achieve rapid haemostasis, they must be used in combination but must be used in conjunction with a meticulous surgical technique. The clips and conventional clamp-and-tie technique ideally are suited to acute bleeding and are most effective when a vessel from a bleeding source can be identified. These surgical sponges are not intended as substitutes of careful surgery, a proper use of sutures and ligatures and must never been used to control haemorrhage from large arteries. Specific experience and training with these new devices is essential for optimal use. Preliminary experience using HPA has been encouraging, but prospective randomized trials using adequate patient numbers are still needed to validate efficacy and safety.

## Competing interests

The author(s) declare that they have no competing interests.

## Authors' contributions

**GD**: acquisition of data

**FR**: study conception and design

**LB**: analysis and interpretation of data

**GD**: drafting of manuscript

**RD**: Critical revision and supervision
